# Cellular phenotype impacts human immunodeficiency virus type 1 viral protein R subcellular localization

**DOI:** 10.1186/1743-422X-8-397

**Published:** 2011-08-10

**Authors:** Adriano Ferrucci, Michael R Nonnemacher, Brian Wigdahl

**Affiliations:** 1 School of Biomedical Engineering, Science and Health Systems, Drexel University, 3141 Chestnut Street, Philadelphia, PA 19104, USA; 2 Department of Microbiology and Immunology, Drexel University College of Medicine, 2900 Queen Lane, Philadelphia, PA 19129, USA; 3 Center for Molecular Virology and Translational Neuroscience, Institute for Molecular Medicine and Infectious Disease, Drexel University College of Medicine, 245 North 15th Street, Philadelphia, PA 19102, USA

**Keywords:** HIV-1, Vpr, localization pattern, phenotype, CD4^+ ^T lymphocytes, monocytic cells, bone marrow progenitor cells, astrocytes

## Abstract

**Background:**

Human immunodeficiency virus type 1 (HIV-1) viral protein R (Vpr) is a virion-associated regulatory protein that functions at several points within the viral life cycle and has been shown to accumulate primarily in the nucleus and at the nuclear envelope. However, most studies have investigated Vpr localization employing cell types irrelevant to HIV-1 pathogenesis. To gain a better understanding of how cellular phenotype might impact HIV-1 Vpr intracellular localization, Vpr localization was examined in several cell lines representing major cellular targets for HIV-1 infection within the peripheral blood, bone marrow, and central nervous system (CNS).

**Results:**

Utilizing a green fluorescent protein-tagged Vpr, we detected Vpr mainly in foci inside the nucleus, at the nuclear envelope, and around the nucleoli, with dispersed accumulation in the cytoplasm of human endothelial kidney 293T cells. No differences were observed in Vpr localization pattern with respect to either the location of the tag (N- or C-terminus) or the presence of other viral proteins. Subsequently, the Vpr localization pattern was explored in two primary HIV-1 target cells within the peripheral blood: the CD4^+ ^T lymphocyte (represented by the Jurkat CD4^+ ^T-cell line) and the monocyte-macrophage (represented by the U-937 cell line). Vpr was found primarily in speckles within the cytoplasm of the Jurkat T cells, whereas it accumulated predominantly intranuclearly in U-937 monocytic cells. These patterns differ from that observed in a bone marrow progenitor cell line (TF-1), wherein Vpr localized mainly at the nuclear envelope with some intranuclear punctuate staining. Within the CNS, we examined two astroglioma cell lines and found that Vpr displayed a perinuclear and cytoplasmic distribution.

**Conclusions:**

The results suggest that the pattern of Vpr localization depends on cellular phenotype, probably owing to interactions between Vpr and cell type-specific host factors. These interactions, in turn, are likely coupled to specific roles that Vpr plays in each cell type within the context of the viral life cycle. Phenotype-specific Vpr localization patterns might also provide an explanation with respect to Vpr secretion or release from HIV-1-infected cells within the peripheral blood and CNS.

## Background

Human immunodeficiency virus type 1 (HIV-1) viral protein R (Vpr) is a multifunctional virion-associated accessory protein [1,2]. In general, Vpr functions early during postentry steps [3] and as a *de novo *synthesized protein following integration of the proviral DNA genome. Following viral entry, Vpr participates as a component of the preintegration complex, in the transport of the reverse-transcribed viral genome to the nucleus [4], and after nuclear import [5] Vpr likely plays a key role in regulating immediate-early HIV-1 gene expression from the integrated proviral template prior to the transition to Tat-driven gene expression [6,7]. The role of Vpr along the viral replication cycle is critical in nondividing cells (such as monocytes) [8-10], whereas it has been shown to be dispensable in T-lymphocytic cells and other cell types [11]. Several reports have defined the role Vpr has in cell cycle arrest at the G_2_/M phase and as an apoptotic-inducing protein by promoting the formation of mitochondrial permeability transition pores in several types of cells [12-14]. The number of functions Vpr serves might depend on several distinct factors, such as intracellular Vpr concentrations, stage of infection and/or disease, route of entry, and the availability of specific host cell factors.

In the past 20 years researchers have investigated the role that specific amino acid residues play in conferring a particular Vpr intracellular localization pattern [15-22]. Others have investigated how specific alterations in these residues could affect functional properties of Vpr relative to the viral life cycle at the single-cell level and/or overall host pathogenesis and disease (reviewed in [23,24]). Most of these studies have explored Vpr localization using different protein tags primarily in readily transfectable eukaryotic cell lines (summarized in Table 1). Vpr was detected mostly inside the nucleus [17,25-27] or at the nuclear envelope [16,18,21,28,29], although small amounts were also detected within the cytoplasm. A recent report has also demonstrated how posttransfection time might affect the specific pattern of Vpr localization [27] under specific conditions, suggesting that cellular proteins play an important role with respect to the intracellular localization of Vpr. In addition, Vpr was shown to shuttle between the nucleus and cytoplasm, thus giving rise to a different pattern of accumulation at different time points [20]. In this respect, Sherman and coworkers demonstrated that a chimeric Vpr coupled to pyruvate kinase localized primarily within the cytoplasm (owing to a longer dwell time in that cellular compartment) but would accumulate in the nucleus after blockage of a nuclear export receptor [20]. Vpr nuclear accumulation has been attributed to specific leucine-rich regions within Vpr alpha helices [18], which partially overlap with a reported nuclear export signal [5,20,22,30]. These amino acid stretches have been shown to be involved in Vpr shuttling between the nucleus and cytoplasm.

**Table 1:** Previous studies within different cell types show different Vpr localization patterns

Cell type	Tag system	Tag end	Other tagged proteins	Time (h)	Localization pattern	Comments	Ref
HeLa	b-Gal	C	--	48	n		[17]
	
	HA	N	--	48	pn		[21]
	
	b-Gal	C	--	38	pn, pef		
	
	FLAG	N	--	24	n, pn, pef		[18]
	
	Untagged	--	--	5	n, pn, pef	VV infection	[60]
	
	Untagged	--	--	48	n		[73]
	
	GFP	N	PK	24	c		[74]
	
	EYFP	N	--	24/48/72	n/n, pef/pef	Time-dependent Vpr localization	[27]
		C	--	24/48/72	n, pef/n, pef/pef		
	
	FLAG	N	--	48	n		[57]
	
	GFP	C	--	18	pn		[29]
	
	GFP	C	--	18	n		[16]
	
	EGFP	N	--	24	n, pef		[58]
		C	--		pn		
			
	mCherry	N	--		pn, pef		
		C	--		pn		
	
	EGFP	N	--	24	n, pef		[26]
		C	--		n, pn		
			
	HA	N	--		n, pef		
	
	FLAG	N	IRES + GFP	36	n		[75]
	
	FLAG	C	PKA, 6His	?	n		[76]
				120	n, pn	Infection	
	
	EGFP	N	--	14	n, pef		[25]
	
	FLAG	N	--	48	pn, pef		[59]
	
	FLAG	N	GST (C)	?	n	Micro-injected	[77]
	
	GFP	N	--	24	n		[78]
	
	GFP	N	IRES	24	n	Transduction	[79]
	
	FLAG, YFP	N	--	36, 48	n		[80]

293T	HA	N	--	24	n		[15]
	
	EGFP	N	--	24	n		[27]
		C	--		pn, pef		
	
	FLAG	N	--	48	pn		[57]
	
	GFP	N	--	?	n, pn, pef		[44]
	
	GFP	N	PK	24	c		[20]

COS-7	GFP	N	--	24	n		[78]
	
	Untagged	N	--	48	n, pn, pef		[81]
	
	FLAG	N	--	?	pn, m		[13]

HepG2	FLAG	N	--	48	n		[57]

MDM	CMV	N	--	24	n, pn	Transduction	[62]
	
	GFP	C	--	6	n		[16]
	
	Alexa-488	N	--	48	n	EC Vpr	[63]

Jurkat	Untagged	--	--	48	n, pn		[61]

Perivascular macrophage	Tg	N	--		n	Tg-Vpr mice	[71]

U-87 MG	ECFP	N	--	24	n, c		[7]

A summary of studies reported in the literature about Vpr localization patterns in different cell lines is reported. Most of the studies have been accomplished in HeLa and 293T cells and results have differed according to the tagging system employed, the posttransfection time point, and/or location of the tag (at either the amino- or carboxyl-terminus). Very few studies have utilized other cell types relevant to HIV-1 pathogenesis. Abbreviations are as follows: CMV: cytomegalovirus, EC: extracellular, ECFP: enhanced cyan fluorescent protein, EYFP, enhanced yellow fluorescent protein, FLAG: DYKDDDDK (proprietary tag, Sigma, St. Louis, MO), β-gal: galactosidase, GFP: green fluorescent protein, GST, glutathione S-transferase, HA: hemagglutinin, IRES: internal ribosomal entry site, PK: pyruvate kinase, PKA: protein kinase A, Tg: transgenic, VV: vaccinia virus. Localization patterns are abbreviated as follows: n: nuclear, pn: perinuclear, pef: punctate extranuclear foci, c: cytoplasmic, m: mitochondrial.

Most of the studies published to date have examined the pattern of Vpr localization and the protein signals involved in guiding the compartmentalization of this important viral protein in cellular targets that have only minimal relevance to HIV infection and disease (primarily HEK 293T, HeLa, and COS-1 cells). Targets were likely selected for experimental use on the basis of their high transfection efficiency and ease of *in vitro *growth. Unfortunately, little is known about Vpr localization pattern in cells predominantly targeted by HIV-1 during the course of disease. Among these, CD4^+ ^T lymphocytes and cells of the monoctye-macrophage lineage account for the majority of the HIV-1-infected cells within the peripheral blood (PB) and peripheral lymph nodes, tissues, and organs [31,32]. In the central nervous system (CNS), microglia [33] and perivascular macrophages [34,35] represent the most commonly encountered productively infected cells; astrocytic cells are often infected but have an altered pattern of virus-specific transcription and much lower levels of viral production during the course of infection [36,37]. Neurons are generally considered refractile to HIV-1 infection [38,39] although they are prone to damage as a result of the neurotoxic properties associated with many of the virus-encoded proteins, including Vpr [29,31,40-42]. With this in mind, the primary objective of this study was to explore how Vpr localization patterns might be altered in cell types representative of cellular targets naturally encountered by HIV-1 during the course of disease. To define how the cellular phenotype may impact Vpr localization pattern, six different human cell lines representative of the main cellular targets for HIV-1 within the PB and CNS were utilized in transient gene delivery and expression studies. For each cell line, transfection studies were performed using a *Zoanthus *green fluorescently tagged HIV-1 NL4-3 Vpr, and intracellular localization patterns were subsequently determined by deconvolution immunofluorescence microscopy. Cumulatively, these studies indicate that cellular phenotype impacts the intracellular localization of HIV-1 Vpr. The physical location of the tag (either at the amino- or carboxyl-terminus) did not influence the pattern of Vpr accumulation within any of the human cell types examined in these studies. The Vpr localization patterns in lymphocytic and promonocytic cell lines were shown to be predominantly cytoplasmic and intranuclear, respectively, and differed from that previously reported within HEK 293T cells [16,18,21,28]. In contrast, in a bone marrow (BM) progenitor cell line Vpr was shown to localize in a pattern similar to that previously observed in 293T cells. This pattern of localization was also observed in two different astroglioma cell lines representative of CNS astrocytes. These results suggest that Vpr may utilize distinct cellular host factors (differentially expressed in various cell types), which may be responsible for the observed differences in intracellular localization and may alter the functional properties of Vpr within a given cellular compartment during the course of HIV-1 disease. This study represents the first to examine Vpr localization pattern in a spectrum of cell lines representative of cellular phenotypes targeted by HIV-1 during interaction with the human host.

## Results

### Vpr localization pattern in human 293T cells

**Figure 1:**
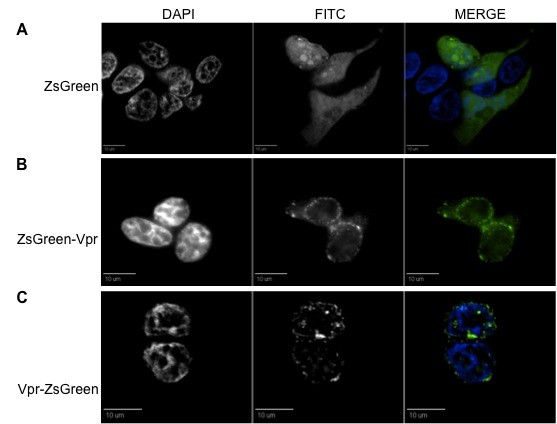
**ZsGreen-tagged Vpr proteins localize at the nuclear envelope and in cytoplasmic speckles within 293T cells**. (A) ZsGreen protein was evenly distributed throughout the cell, with no preference of accumulation in any intracellular organelle. (B) ZsGreen-Vpr and (C) Vpr-ZsGreen show preferential accumulation at the perinuclear boundaries and punctate intranuclear accumulation. Dispersed presence was detectable in the cytoplasm along with localized herniations at the nuclear envelope. The position of the tag (either at the N- or C-terminus) did not influence the Vpr localization pattern. For all figures, the first column represents DAPI (nuclear) staining, the second column represents the FITC (green) channel, and the third column is the merged view where the two channels overlap, in order to give a complete view of the intracellular localization. All figures are shown as cross-sectional views and are representative of the entire cell population observed.

**Figure 2:**
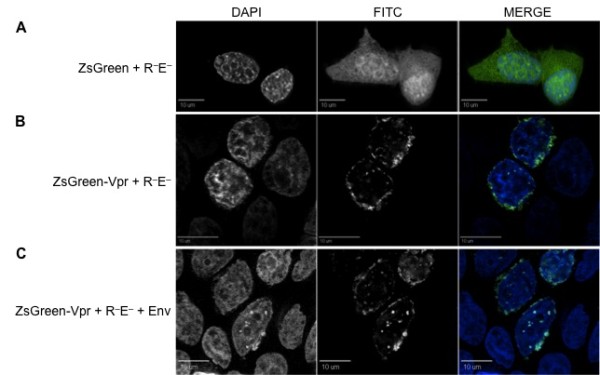
**Cotransfection with other HIV-1 viral proteins does not change Vpr localization pattern within 293T cells**. (A) The ZsGreen expression vector cotransfected with a Vpr- and Env-deficient (pNL4-3R^-^E^-^) molecular clone maintains its uniform ZsGreen localization throughout the intracellular environment. The ZsGreen-Vpr expression vector cotransfected with pNL4-3R^-^E^- ^molecular clone without (B) or with the envelope protein (C) preserves the same localization pattern as ZsGreen-Vpr (Fig. 1). The first, second, and third columns represent DAPI, FITC, and merged views, respectively. Each figure represents a cross-sectional view of the cell and is representative of the entire cell population observed.

**Figure 3:**
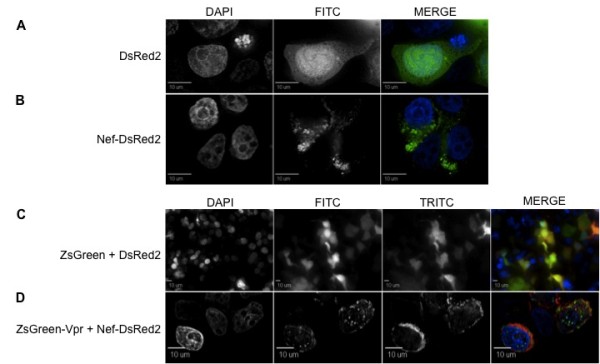
**ZsGreen-Vpr localization pattern in 293T cells is not affected by addition of Nef protein**. (A) DsRed2, similar to ZsGreen protein, was uniformly distributed throughout the intracellular environment. (B) Nef-DsRed2 displays a cytoplasmic accumulation, being completely excluded from the nuclear compartment. (C) As a control, ZsGreen and DsRed2 expression vectors were cotransfected and both localized evenly in the intracellular environment (as evidenced by the overlapping yellow staining). (D) When both ZsGreen-Vpr and Nef-DsRed2 expression vectors were cotransfected, neither protein's localization pattern (perinuclear and cytoplasmic, respectively) was altered. In parts A and B, the first, second, and third columns are DAPI, FITC, and merged view, respectively. In parts C and D, the first, second, third, and fourth columns represent DAPI, FITC (green), TRITC (red), and merged views, respectively. Each figure represents a cross-sectional view of the cell and is representative of the entire cell population observed.

Previous studies have examined localization patterns of Vpr in full-length form, shorter cleaved forms, as Vpr peptides, or as Vpr variants with alternative amino acid residues at specific positions deemed important in guiding Vpr nuclear localization (Table 1). The choice of the cell type used for each localization study was often dictated by a desire to achieve an optimal gene delivery efficiency to facilitate examination of specific Vpr functional properties. Relevant to this experimental strategy, previous studies have suggested that cellular phenotype may alter the requirement for Vpr during the course of productive viral replication [8-11]. On the basis of these observations, we hypothesized that cellular phenotype and hence specific interactions with selected cellular proteins alter the pattern of Vpr localization. In this regard, we examined the intracellular localization of Vpr following transfection of a spectrum of cellular targets representative of cell populations during the course of HIV-1 disease. To this end, we have used as a tagging system a *Zoanthus *green (ZsGreen) fluorescent protein, which has been previously shown to have higher intracellular stability than enhanced green fluorescent protein (EGFP) [43]. For comparative purposes, the localization of each Vpr construct was first examined in HEK 293T cells. Cells were transfected by the calcium phosphate method and intracellular localization was evaluated by deconvolution immunofluorescence microscopy at 24 h after transfection. While ZsGreen protein does not show preferential localization (Figure 1A), Vpr tagged with ZsGreen either at the N-terminus (ZsGreen-Vpr) or C-terminus (Vpr-ZsGreen) (Figure 1B and 1C, respectively) was located primarily at the nuclear envelope, as well as in speckles within the nucleus and cytoplasm. These findings confirm previous Vpr localization studies in this cell type [27,44,45], unlike a study wherein EGFP-Vpr and Vpr-EGFP localization patterns were found to differ in HEK 293T cells [27]. In addition, Vpr-induced localized herniations were observed at the nuclear envelope, as previously reported [46]. We next examined whether other HIV-1 proteins could affect Vpr localization as a more representative scenario of *in vivo *HIV-1 infection. To this end, 293T cells were cotransfected with the Vpr- and Env-deficient pNL4-3R^-^E^- ^molecular clone along with the ZsGreen-Vpr plasmid. Localizations of the ZsGreen protein alone as well as that of ZsGreen-Vpr were not perturbed by the presence of the viral proteins expressed from the molecular clone (Figure 2A and 2B). Also, the addition of the envelope protein in the cotransfection reaction did not alter Vpr localization in 293T cells (Figure 2C). Because the molecular clone utilized in these studies was also Nef-deficient, the presence of this viral protein was also assessed to determine whether it would have any impact on Vpr localization. While DsRed2 was uniformly distributed intracellularly (Figure 3A), Nef-DsRed2 was found primarily within the cytoplasm (Figure 3B), which confirms previously reported observations [47-49]. Because both Vpr and Nef have been cloned in frame with two different color-emitting proteins, studies were performed to confirm that neither of the fluorescently labeled proteins alters the localization pattern of the other protein. Indeed, both ZsGreen and DsRed2 are evenly present throughout the intracellular compartment (Figure 3C). Cotransfection of ZsGreen-Vpr and Nef-DsRed2 clearly demonstrated that both viral proteins maintain their typical localization patterns (Figure 3D), without interfering with the localization of the other protein. These results prompted us to further investigate possible variations in the intracellular localization of only the HIV-1 Vpr protein without any other viral proteins cotransfected in *cis *or in *trans *with the Vpr-containing plasmid.

### Vpr localization pattern in human blood-borne cells

**Figure 4:**
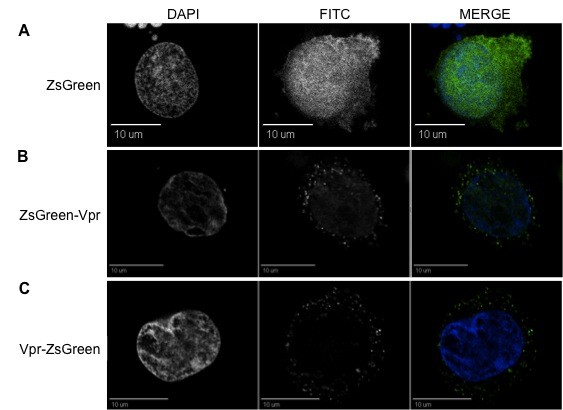
**ZsGreen-Vpr localizes exclusively in the cytoplasm within Jurkat lymphocytic cells**. While control ZsGreen protein (A) was evenly distributed, both ZsGreen-Vpr (B) and Vpr-ZsGreen (C) were found in speckles only within the cytoplasmic compartment, completely excluded from the nucleus. First, second, and third columns are DAPI, FITC, and merged views, respectively. Each figure represents a cross-sectional view of the cell and is representative of the entire cell population observed.

**Figure 5:**
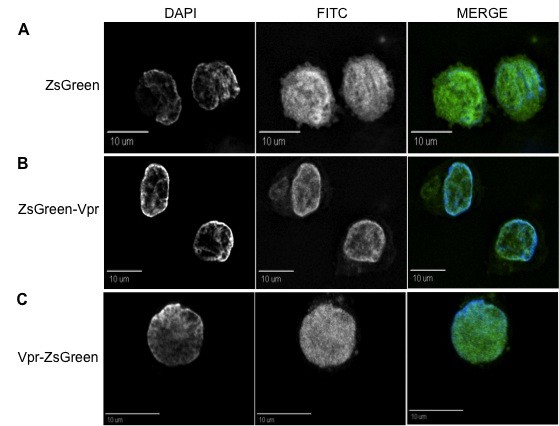
**Localization of ZsGreen-Vpr within U-937 promonocytic cells is predominantly intranuclear**. (A) ZsGreen control protein localizes uniformly intracellularly, while both ZsGreen-Vpr (B) and Vpr-ZsGreen (C) are similarly distributed within the nucleus, with a very low level accumulation in the cytoplasm, unlike Jurkat cells. The first, second, and third columns represent DAPI, FITC, and merged views, respectively. All figures denote a cross-sectional view of the cell. Similar results have been obtained in triplicates.

**Figure 6:**
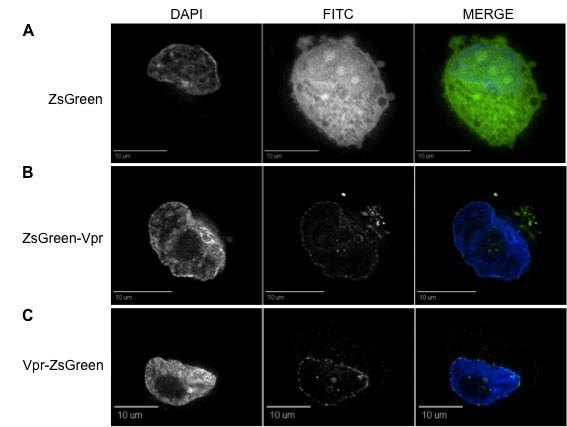
**ZsGreen-tagged Vpr localizes at the perinuclear rim within TF-1 bone marrow progenitor cells**. (A) ZsGreen protein localized uniformly intracellularly (including nucleoli). (B and C) Both ZsGreen-Vpr and Vpr-ZsGreen display an intracellular localization pattern similar to that observed in HEK 293T cells. Vpr is found primarily at the nuclear envelope, although a low level of accumulation was also found in speckles both in the nucleus and in the cytoplasm. Similar to 293T cells, localized herniations were observed at the nuclear rim. The first, second, and third columns represent DAPI, FITC, and merged views, respectively. All figures denote a cross-sectional view of the cell and are representative of the entire cell population.

T cells and cells of the monocyte-macrophage lineage present in the human PB and regional lymph nodes are commonly infected by HIV-1 during the course of HIV/AIDS and constitute the majority of the HIV-1-infected cells in these compartments. In this regard, the Jurkat and U-937 human cell lines have been used as cells representative of CD4^+ ^T lymphocytes and monocytic cells. As shown in Figure 4, ZsGreen uniformly localized throughout the intracellular environment of Jurkat cells (Figure 4A), whereas both N- and C-terminally ZsGreen-tagged Vpr proteins were found to accumulate exclusively as foci within the cytoplasm (Figure 4B and 4C). In contrast, when we analyzed U-937 cells, while ZsGreen protein alone maintained an even distribution intracellularly (Figure 5A), both ZsGreen-Vpr and Vpr-ZsGreen were distributed predominantly inside the nucleus, with a small amount of Vpr dispersed within the cytoplasm (Figure 5B and 5C). Similar to HEK 293T cells, the location of the tag does not alter the pattern of Vpr localization. However, the localization pattern is remarkably different among these three cell types: (1) mainly around the nuclear envelope (293T), (2) intranuclear (U-937), and (3) punctate cytoplasmic (Jurkat). Because all blood-borne cells are derived from the BM, studies were designed to determine how Vpr distributed within a progenitor cell population to ascertain whether there are basic differences in Vpr accumulation in a cellular phenotype similar to that encountered in the early stages of BM differentiation. As one approach to examine this question, the TF-1 human BM progenitor cell line was transfected with ZsGreen-tagged Vpr constructs as described in "Methods." TF-1 cells have been shown to exhibit two morphologies: a more frequently encountered round-shaped one similar to lymphocytes or monocytes and a less frequent elongated one, comparable to a fibroblastic morphology. In TF-1 cells, ZsGreen protein was present throughout the cellular compartment (Figure 6A), whereas both types of Vpr were distributed prevalently at the nuclear rim, with scarce speckled structures found both intranuclearly and within the cytoplasm (Figure 6B and 6C). This localization pattern was observed regardless of the observed cellular morphology (round or elongated) (data not shown).

### Vpr localization pattern in human astroglial cells

**Figure 7:**
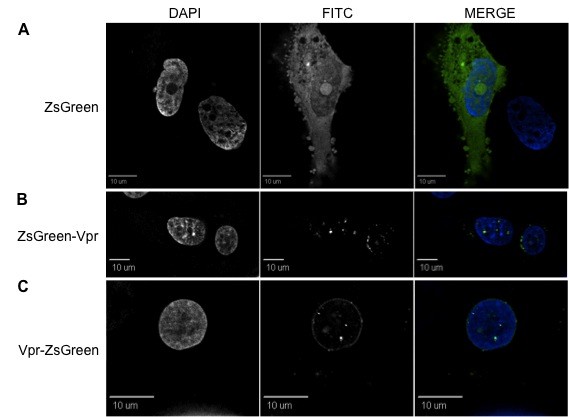
**In U-373 MG astroglioma cells, ZsGreen-Vpr shows a localization pattern similar to HEK 293T cells**. (A) While ZsGreen protein clearly distributed throughout the intracellular environment, both ZsGreen-Vpr (B) and Vpr-ZsGreen (C) were found mainly at the nuclear envelope, along with localized herniations around the nuclear membrane, and in speckles within the nuclear compartment. First, second, and third columns are DAPI, FITC, and merged views, respectively, and each figure represents a cross-sectional view of the cell. These figures are representative of the entire cell population observed.

**Figure 8:**
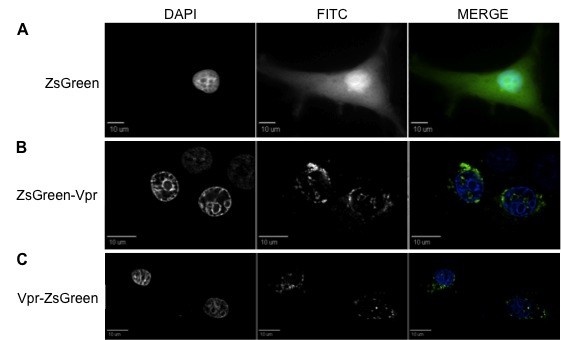
**U-87 MG displays a localization pattern of ZsGreen-tagged Vpr similar to U-373 MG astroglial cells**. ZsGreen protein was uniformly distributed intracellularly. ZsGreen-Vpr (B) and Vpr-ZsGreen (C) localized at the nuclear envelope, along with a speckled formation intranuclearly. An accumulation around the nucleolar rim could also be observed, as well as localized herniations at the nuclear envelope. A conspicuous amount was also noticeable at the immediate outer layer of the nuclear envelope, possibly coinciding with the endoplasmic reticulum. The first, second, and third columns represent DAPI, FITC, and merged views, respectively. All figures denote a cross-sectional view of the cell and are representative of the entire cell population.

In addition to the PB and peripheral lymphoid tissues, HIV-1-infected cells have been shown to increase in a number of end organs such as the mucosal tissues, lungs, kidneys, and the brain [50-54]. Among CNS-resident cells, microglia and infiltrating monocytes are the cells most commonly found to be productively infected by HIV-1 [34,35,55], although recently the role of astrocytes has been reconsidered owing not only to their abundance but also to an underestimation of their infection rate [36,37]. Neurons, although sensitive to both HIV-1-induced and indirect effects, are still considered to be refractile to infection by the virus. Owing to the absence of an ideal human microglial cell line and to the increasing evidence of the importance of astrocytes in HIV-1-related neuropathogenesis, we focused our attention on astrocytic cells and investigated HIV-1 Vpr localization in two astroglioma cell lines: U-373 MG and U-87 MG. Nucleofection studies show that while ZsGreen preserves a global uniform distribution in both cell lines (Figures 7A and 8A), ZsGreen-Vpr and Vpr-ZsGreen are found primarily at the nuclear envelope and around nucleoli, although a distinct presence is noticeable around the nuclear rim, probably coinciding with the endoplasmic reticulum (Figure 7B and 7C and Figure 8B and 8C). In addition, typical localized herniations could be observed at the nuclear envelope similar to those previously observed in 293T cells.

## Discussion

HIV-1 Vpr is a viral regulatory protein that acts at many points along the viral life cycle. Vpr intracellular localization and dwell time in different compartments depend on the intrinsic molecular interactions exerted between distinctive Vpr domains and other viral or cellular partner proteins (for a review, see [56]). These interactions depend not only on the stage of the viral life cycle but also on the expression of intracellular binding partners, which are ultimately differentially regulated in selected cellular phenotypes. Indeed, HIV-1 Vpr has been shown to be essential to productively infect cells of the monocyte-macrophage lineage [8-10], whereas its presence is dispensable for replication within lymphocytes [11]. This could suggest the presence of different host cellular factors differentially expressed in the two cell types, which Vpr relies upon, or of negative regulatory factors in lymphocytes (absent in monocytes) that eliminate the requirement for Vpr with respect to productive replication.

In the studies described herein, different cellular microenvironments were examined for their potential impact on the pattern of Vpr localization observed under selected experimental conditions. To this end, six different cell lines representative of important HIV-1 targets within the PB, BM, and CNS were explored. We determined that the location of the ZsGreen-emitting protein at either the N- or C-terminus did not affect Vpr localization in HEK 293T cells, unlike a previously reported study performed with the EGFP [27]. Indeed, as reported by several independent studies [18,44,57-60], Vpr is found predominantly at the nuclear rim and to a lesser extent in speckles present both inside the nucleus and within the cytoplasm. In addition, localized herniations were detected at the nuclear envelope in HEK 293T cells, as has been previously reported by other investigators [46]. The herniated areas may facilitate shuttling or leakage of Vpr molecules between the nuclear and cytoplasmic compartments. To model a more relevant *in vivo *scenario, studies are underway to determine whether the presence of the other HIV-1 viral proteins could influence Vpr localization by cotransfection studies. The results have clearly indicated that none of the other viral proteins, added either singly or in combination, altered the intracellular location of Vpr. Studies then proceeded to examine Vpr localization of Vpr within the two human cell lines representative of the main cellular targets for HIV-1 within the PB. In this respect, our studies clearly demonstrated that the CD4^+ ^T-lymphocytic Jurkat cells exhibited primarily a cytoplasmic localization consisting of punctate cytoplasmic foci. These results partly contrast with published results employing a Jurkat cell line [61], although with a different clone than the one utilized in the present study. In this regard, Bolton and coworkers demonstrated primarily nuclear localization of Vpr, with small amounts of Vpr localized to the cytoplasm, although other cells within the same population showed a more perinuclear localization pattern. However, the difference between the two studies may relate to the difference in the posttransfection time at which cells were analyzed (24 h after transfection herein as compared with 48 h in the previous study [61]), although in an additional study, a Vpr peptide (residues 52-96) added extracellularly to lymphocytes and lymphoblasts was distributed exclusively within the cytoplasm [13]. In contrast to T cells, within a promonocytic U-937 cell line, Vpr appears to accumulate exclusively within the nucleus, as also shown in one study employing transfected monocyte-derived macrophages [16]. Two additional independent studies have also found a nuclear pattern in monocyte-derived macrophages either transduced by a Vpr-containing adenovirus vector [62] or treated with an extracellular synthetic Vpr [63].

Hematopoietic differentiation occurs within the BM, after which time cells leave the BM and eventually enter the peripheral circulation. As one approach to model this developmental process, Vpr localization studies were performed in a human CD34^+ ^BM progenitor cell line to determine whether this was similar to the pattern observed in either differentiated cell type (lymphocytes or monocytes). TF-1 cells showed perinuclear localization, with accumulation involving punctate foci both within the nucleus and likely at the endoplasmic reticulum, a pattern of accumulation more similar to that observed in 293T cells than either the lymphocytic or monocytic cell line. These observations suggest that cellular differentiation and/or activation results in changes in the pattern of Vpr accumulation. These changes may be closely linked to differentiation- and/or state-of-activation-dependent alterations in the presence or modification of intracellular factors regulated during the differentiation process. Indeed, the Vpr localization pattern in TF-1 was clearly different from that found within representative cell lines of CD4^+ ^T-lymphocytic or monocytic origin.

The CNS, although anatomically compartmentalized, is surveyed by cells involved in immune surveillance and is also breached by potentially neurotoxic substances and/or cells infected by any of a number of infectious agents, including HIV, particularly under circumstances of pathogenic insult. Within the CNS, HIV-1 preferentially targets microglia, trafficking monocyte-derived macrophages and brain microvascular endothelial cells, and, to a lesser extent, astrocytes, which likely represent an important CNS reservoir simply based on their numbers. Owing to the lack of a reliable microglial cell line, we have investigated two different types of astroglioma cell lines (U-373 MG and U-87 MG) and found that Vpr displayed a similar localization pattern in both. A predominant accumulation around the nuclear envelope was observed, although Vpr also localized within the cytoplasm and to a lesser extent in the nucleus and around the nucleoli. This localization pattern was most similar to that observed in one previous study performed in U-87 MG cells [7].

The differential localization patterns we observed in the aforementioned cell lines indicate that the cellular phenotype affects the intracellular distribution of Vpr and point to a role that Vpr might play in each cell type. In this regard, a nuclear localization pattern in monocyte-derived macrophages may reflect a role Vpr could have as a transcription factor. This possibility is substantiated by studies that confirmed a lack (or low level) of infectivity for Vpr-deficient molecular clones in cells of the monocytic lineage. Conversely, a cytoplasmic accumulation in CD4^+ ^T lymphocytes could denote a more active participation in later phases of the viral cycle, such as virion incorporation or as a secreted protein. In the former case, the presence of Vpr within the cytoplasm could reflect an increased interaction with p6 proteins as more viral transcription takes place. Indeed, within CD4^+ ^T cells, Vpr is known to synergize with Nef in activating nuclear factor of activated T cells (NFAT)-dependent and cyclic AMP response element (CRE)-directed transcription [64]. This leads to increased viral genome production owing to the presence of NFAT and CRE-responsive elements within the HIV-1 promoter. In the latter case, because Vpr has been shown to be released from Jurkat cells [65] and extracellular Vpr has been detected within the blood of HIV-1-infected patients [66,67], this cell type could be responsible for secretion of Vpr within the PB compartment, given the Vpr cytoplasmic distribution in this T-cell line. However, it remains possible that Vpr release is just the consequence of Vpr's apoptogenic nature rather than the result of an active secretion process (for a review, see Ferrucci et al., 2011, manuscript in press). A further explanation of the different intracellular localization of Vpr between Jurkat and U-937 cells could lie in the differential expression of Vpr binding partners between the two cell lines. For instance, Vpr is known to interact with UNG, a uracil DNA glycosylase repair enzyme [68], whose gene gives rise to UNG1 and UNG2 by alternative splicing, found cytoplasmically and nuclearly, respectively [69]. A possible phenotype-specific differential expression of either form in the two cell lines, such that UNG1 were found predominantly in Jurkat (or CD4^+ ^T lymphocytes) and UNG2 primarily in U-937 (or monocytic cells), could then explain why Vpr localizes within the cytoplasm or the nucleus, respectively. Other important intracellular host factors Vpr has been shown to interact with are the Sp1 and CCAAT enhancer binding proteins (C/EBP) [70], which are differentially regulated in several different cell lines. For instance, the overabundance of C/EBP in U-937 monocytic cells compared with Jurkat lymphocytes (Y. Liu and B. Wigdahl, unpublished observations) could explain a more nuclear localization of Vpr in the former cell line. Conversely, a more perinuclear localization in the other cell lines examined could be the cause of decreased affinity with the aforementioned proteins, rather a stronger binding for factors such as importin-α for which Vpr was shown to interact [4,18,21].

Within the CNS, infected infiltrating CD4^+ ^T lymphocytes and trafficking monocytes play an important role in HIV-1 pathogenesis by continually seeding virus into the CNS. Because Vpr has been found in a soluble form within the cerebrospinal fluid of HIV-1-infected patients [67], these two cell types could participate in the release process both within the PB and CNS compartments. In this regard, after either lymphocytes or monocytes infected with HIV-1 and expressing Vpr have crossed the blood-brain barrier it is possible that intra-CNS cytokines or other signaling molecules induce changes in the levels and activities of intracellular factors. In turn, these may play a role in altering the intracellular accumulation of Vpr, which ultimately leads to functional changes in these infected cell populations in ways that may alter the course of HIV-1-associated neurologic disease. In addition, given the extensive amount of Vpr in the cytoplasm of astrocytes and because of their number within the CNS, these cells also contribute to Vpr release either through a regulated secretory process or through necrosis or apoptosis. Among other CNS-resident cells, brain perivascular and parenchymal microglial cells could also contribute to seeding viral infection within the CNS. Only one study has been published that showed a predominant nuclear localization of Vpr in these two cell types, although the study was performed in Vpr-transgenic mice [71].

## Conclusion

The present study is the first to extensively analyze and compare Vpr localization in different cell types relevant to HIV-1 infection. These results summarize and amplify the breadth of knowledge available to date concerning Vpr intracellular localization and impact on HIV-1 pathogenesis (Table 1). We observed different localization patterns in different cell types that represent primary targets of HIV-1, which may relate to differences in Vpr function within each cell type. Future studies employing different primary HIV-1 target cells are needed to clearly discern how cellular phenotypes affect Vpr intracellular trafficking. However, it must be asserted that most of the reported studies, as well as the studies reported herein, have employed transfection methods, which generally involved overproduction of the desired protein. This scenario more closely reflects an acute cellular infection, with abundant protein synthesis and viral production. It will be informative to examine Vpr localization at different time points in cells infected with Vpr-containing virus, as this would more accurately model the *in vivo *environment. This type of model would also facilitate studies to determine how the recognition of specific amino acid residues and localization signals could be altered in different cell types. For instance, nuclear localization signals could be masked in different cellular environments by interactions with host cell factors not present in other cells. It is indeed possible to hypothesize that organelle localization signals might be cell type-dependent, owing either to a masking effect or to different protein-protein interactions in distinct cell types. Another approach would be to employ a cell line stably transfected with a Vpr-expressing plasmid and analyze how Vpr localization pattern varies during latency or after administration of stimuli known to rescue productive viral replication.

## Methods

### Cell lines

The human cell lines utilized in the protein localization studies included the human endothelial kidney (HEK) 293T (fibroblast), Jurkat (CD4^+ ^T cells), U-937 (promonocytic), U-87 MG and U-373 MG (both of astroglioma origin), and TF-1 (BM progenitor). The HEK 293T cell line was grown in 1 × Dulbecco modified Eagle medium (DMEM) supplemented with heat-inactivated fetal bovine serum (FBS) (10%), antibiotics (penicillin and streptomycin, at a concentration of 0.04 mg/mL each), glucose (4.5 g/mL), sodium pyruvate (1 mM), and HEPES (10 mM). The human T-cell line (Jurkat), the human monocytic cell line (U-937), and the erythromyeloid precursor cell line (TF-1) were all grown in RPMI-1640 (Roswell Park Memorial Institute) media. Media for Jurkat cells were supplemented with heat-inactivated FBS (10%), sodium bicarbonate (0.05%), and antibiotics (penicillin, streptomycin, and kanamycin at 0.04 mg/mL each). Media for U-937 and TF-1 cells were supplemented with heat-inactivated FBS (10%), antibiotics (penicillin and streptomycin, at a concentration of 0.04 mg/mL each), glucose (4.5 g/mL), sodium pyruvate (1 mM), and HEPES (10 mM). TF-1 cells were also supplemented with recombinant granulocyte-macrophage colony-stimulating factor (GM-CSF) (2 ng/mL). The human astroglioma cell lines (U-87 MG and U-373 MG) were grown in 1 × DMEM supplemented with heat-inactivated FBS (10%), penicillin and streptomycin (at a concentration of 0.04 mg/mL each), kanamycin sulfate (0.04 mg/mL), and sodium bicarbonate (final concentration 0.05%). All cells were maintained at 37°C in 5% CO_2 _at 90% relative humidity.

### Construction of the plasmids

HIV-1 Vpr was obtained by standard PCR amplification techniques from the noninfectious pNL4-3R^+^E^- ^molecular clone (obtained through the NIH AIDS Research and Reference Reagent Program, Division of AIDS, NIAID, NIH, provided by Dr. Nathaniel Landau [8,55]). To clone ZsGreen-Vpr and Vpr-ZsGreen, pZsGreen-C1 and pZsGreen-N1 vectors (Clontech, Mountain View, CA) were used to place the green fluorescent protein at either the amino- or carboxyl-terminus of the viral protein, respectively. The following PCR primers were employed: 5'-GATCTCGAGCTATGGAACAAGCCCCAGAAGACC and 5'-GACGTGAAGCTTCTAGGATCTACTGGCTCC (forward and reverse to clone ZsGreen-Vpr) and 5'-ATAGGATCC**GCCACC**ATGGAACAAGCCCCAGAAGACC and 5'-CGAACCGGTCCACCTGATCTACTGGCTCC (forward and reverse to clone Vpr-ZsGreen). The different primers harbor specifically engineered restriction endonuclease cleavage sites as underscored in each of the sequences provided. Forward and reverse primers to clone ZsGreen-Vpr contain *Xho*I and *Hin*dIII cleavage sites, respectively, whereas forward and reverse primers for Vpr-ZsGreen include *Bam*HI and *Age*I, respectively. The forward primer to the PCR construct (Vpr-ZsGreen) also incorporated a consensus Kozak sequence (in bold) prior to the Vpr ATG to aid in initiation of the translation process [72]. PCR-amplified Vpr products and ZsGreen-containing vectors were then digested with the respective restriction endonucleases (Promega, Madison, WI) in a one-step reaction for 1 h at 37°C to obtain complementary single-stranded sequences at the ends of each product. Cloning of Vpr products and ZsGreen-containing vectors was then completed by overnight ligation at 4°C with T4 DNA ligase (Promega). The pNL4-3R^-^E^- ^molecular clone (with a four-basepair insertion to inactivate the Vpr coding sequence) was obtained as described for pNL4-3R^+^E^-^. Because both the pNL4-3R^+^E^- ^and pNL4-3R^-^E^- ^molecular clones are also Nef-deficient owing to the insertion of a luciferase gene, a Nef-containing plasmid (kindly provided by Dr. Simon Cocklin, Drexel University College of Medicine, Philadelphia, PA) was utilized to clone Nef into the pDsRed2-N1 expression vector (Clontech). Forward (5'-ATAAAGCTT**GCCACC**ATGGGAGGGAAGTGG) and reverse (5'-GATGGATCCCCTCCGCAGTTCTTGAAGTACTCC) primers were used to PCR amplify Nef, which include *Hin*dIII and *Bam*HI cleavage sites, respectively. The forward primer also contains a consensus Kozak sequence at the 5' end of the ATG transcriptional start site. The PCR products and pDsRed2-N1 plasmid were then digested with *Hin*dIII and *Bam*HI restriction enzymes (Promega) and ligated overnight at 4°C as described for the Vpr-containing recombinant plasmids. The envelope-containing plasmid was a kind gift from Dr. Julio Martin-Garcia (Drexel University College of Medicine, Philadelphia, PA). All cloned constructs were confirmed by DNA sequencing (Genewiz, Inc., South Plainfield, NJ).

### Transfection studies

Exponentially growing HEK 293T cells (2 × 10^5^) were seeded in each well of a 12-well plate. Four hours prior to transfection, the cell medium was replaced and transfections were performed at about 70% cell confluency with the classical calcium phosphate method using 2 μg of plasmid DNA (either ZsGreen control or Vpr plasmid) mixed with CaCl_2 _to a final concentration 60 mM. The CaCl_2_-DNA mixture was then added dropwise into a solution of 2× HEPES-buffered saline (HBS). Following a 30-min incubation at room temperature, the CaCl_2_-DNA-2× HBS mixtures were dispensed dropwise to the cell culture monolayers. After a 5-h incubation, the cell culture medium was replaced and the cell monolayers were incubated for an additional 24 h prior to processing for immunofluorescent microscopic analyses. For cotransfection studies, the DNA mixture was a combination of Vpr, pNL4-3R^-^E^- ^molecular clone, and envelope and Nef plasmids in a 1:2:1:1 ratio. The calcium phosphate transfection method consistently produced a 50% transfection efficiency.

For all other cell lines, transfection was performed using a chemical/electroporation technique (nucleofection) as described by the manufacturer (Amaxa; Lonza, Basel, Switzerland). Briefly, 2 days prior to transfection, 10^6 ^suspension cells (Jurkat, U-937, and TF-1 cells), passaged to allow them a logarithmic phase growth on the day of transfection, were washed twice in 1× phosphate-buffered saline (PBS) and then resuspended in 100 μL nucleofector solution containing 2 μg of plasmid DNA. The mixture was then placed into an appropriate cuvette, the cells nucleofected using the proprietary Amaxa device and transferred back to a single well of a 12-well plate. For the two adherent astroglioma cell lines, the same protocol was used with the addition of an initial trypsinization step prior to resuspension. Each cell line was transfected with a different nucleofection procedure and protocol program as follows: Jurkat (procedure V, program X-005), U-937 (procedure V, program W-001), TF-1 (procedure T, program T-001), and two astroglioma cell lines (procedure T, program X-001). Overall, this nucleofection method yielded a delivery efficiency of 30-40% for both astroglioma cell lines and TF-1 cells, whereas Jurkat and U-937 cells yielded a delivery efficiency of about 5%.

### Immunofluorescence and deconvolution microscopy

HEK 293T cells were grown on poly-lysine-coated coverslips and transfected *in situ*, whereas all other cells were first nucleofected and then transferred to poly-lysine-coated coverslips immediately after transfection. For all immunofluorescence studies, cells were harvested 24 h after transfection, washed twice with PBS, and fixed in 2% paraformaldehyde for 20 min. Cells were then stained with 4',6-diamidino-2-phenylindole (DAPI; Invitrogen, Carlsbad, CA) and mounted on slides. Cells were washed three times in between each step and imaged with a deconvolution microscope (Olympus, Center Valley, PA). For all cell types examined, a minimum of 10 fields were examined at 40× magnification. Images shown at higher magnifications are representative of the entire cell population with a minimum of 20 different sectioned planes examined at a distance of 0.2 μm and representative images captured for the different channels (DAPI, fluorescein isothiocyanate [FITC], and/or tetramethylrhodamine isothiocyanate [TRITC]). Deconvolution using the nearest-neighbor algorithm was applied to all images in order to deblur scattered light from neighboring planes and improve the image contrast. Deconvolved planes were also stacked to yield a three-dimensional reconstruction of volume images in order to confirm Vpr localization.

## List of abbreviations

BM: bone marrow; C/EBP: CCAAT enhancer binding proteins; CNS: central nervous system; CRE: cyclic AMP response element; DAPI: 4',6-diamidino-2-phenylindole; DMEM: Dulbecco modified Eagle medium; EGFP: enhanced green fluorescent protein; FBS: fetal bovine serum; FITC: fluorescein isothiocyanate; FLAG: DYKDDDDK (proprietary tag, Sigma, St. Louis, MO); GM-CSF: granulocyte-macrophage colony-stimulating factor; HA: hemagglutinin; HBS: HEPES-buffered saline; HEK: human endothelial kidney; HIV-1: human immunodeficiency virus type 1; NFAT: nuclear factor of activated T cells; PB: peripheral blood; PBS: phosphate-buffered saline; RPMI: Roswell Park Memorial Institute; TRITC: tetramethylrhodamine isothiocyanate; Vpr: viral protein R (regulatory); ZsGreen: *Zoanthus *green emitting protein.

## Competing interests

The authors declare that they have no competing interests.

## Authors' contributions

AF was the principal experimentalist who was involved in the conception of this study, collected and analyzed data, and prepared the first draft of the manuscript. MRN and BW participated in the design and coordination of this study and critically reviewed the manuscript. All authors read and approved the final manuscript.

## References

[B1] Cohen EA Dehni G Sodroski JG Haseltine WA Human immunodeficiency virus vpr product is a virion-associated regulatory protein J Virol 1990 64 3097 3099 2139896 10.1128/jvi.64.6.3097-3099.1990 PMC249501

[B2] Paxton W Connor RI Landau NR Incorporation of Vpr into human immunodeficiency virus type 1 virions: requirement for the p6 region of gag and mutational analysis J Virol 1993 67 7229 7237 8230445 10.1128/jvi.67.12.7229-7237.1993 PMC238185

[B3] Hrimech M Yao XJ Bachand F Rougeau N Cohen EA Human immunodeficiency virus type 1 (HIV-1) Vpr functions as an immediate-early protein during HIV-1 infection J Virol 1999 73 4101 4109 10196306 10.1128/jvi.73.5.4101-4109.1999 PMC104189

[B4] Popov S Rexach M Zybarth G Reiling N Lee MA Ratner L Lane CM Moore MS Blobel G Bukrinsky M Viral protein R regulates nuclear import of the HIV-1 pre-integration complex EMBO J 1998 17 909 917 10.1093/emboj/17.4.909 9463369 PMC1170440

[B5] Sherman MP de Noronha CM Eckstein LA Hataye J Mundt P Williams SA Neidleman JA Goldsmith MA Greene WC Nuclear export of Vpr is required for efficient replication of human immunodeficiency virus type 1 in tissue macrophages J Virol 2003 77 7582 7589 10.1128/JVI.77.13.7582-7589.2003 12805458 PMC164827

[B6] Hogan TH Nonnemacher MR Krebs FC Henderson A Wigdahl B HIV-1 Vpr binding to HIV-1 LTR C/EBP cis-acting elements and adjacent regions is sequence-specific Biomed Pharmacother 2003 57 41 48 10.1016/S0753-3322(02)00333-5 12642036

[B7] Cui J Tungaturthi PK Ayyavoo V Ghafouri M Ariga H Khalili K Srinivasan A Amini S Sawaya BE The role of Vpr in the regulation of HIV-1 gene expression Cell Cycle 2006 5 2626 2638 10.4161/cc.5.22.3442 17172832

[B8] Connor RI Chen BK Choe S Landau NR Vpr is required for efficient replication of human immunodeficiency virus type-1 in mononuclear phagocytes Virology 1995 206 935 944 10.1006/viro.1995.1016 7531918

[B9] Subbramanian RA Kessous-Elbaz A Lodge R Forget J Yao XJ Bergeron D Cohen EA Human immunodeficiency virus type 1 Vpr is a positive regulator of viral transcription and infectivity in primary human macrophages J Exp Med 1998 187 1103 1111 10.1084/jem.187.7.1103 9529326 PMC2212198

[B10] Nitahara-Kasahara Y Kamata M Yamamoto T Zhang X Miyamoto Y Muneta K Iijima S Yoneda Y Tsunetsugu-Yokota Y Aida Y Novel nuclear import of Vpr promoted by importin alpha is crucial for human immunodeficiency virus type 1 replication in macrophages J Virol 2007 81 5284 5293 10.1128/JVI.01928-06 17344301 PMC1900242

[B11] Eckstein DA Sherman MP Penn ML Chin PS De Noronha CM Greene WC Goldsmith MA HIV-1 Vpr enhances viral burden by facilitating infection of tissue macrophages but not nondividing CD4+ T cells J Exp Med 2001 194 1407 1419 10.1084/jem.194.10.1407 11714748 PMC2193684

[B12] Jacotot E Ferri KF El Hamel C Brenner C Druillennec S Hoebeke J Rustin P Metivier D Lenoir C Geuskens M Control of mitochondrial membrane permeabilization by adenine nucleotide translocator interacting with HIV-1 viral protein rR and Bcl-2 J Exp Med 2001 193 509 519 10.1084/jem.193.4.509 11181702 PMC2195906

[B13] Jacotot E Ravagnan L Loeffler M Ferri KF Vieira HL Zamzami N Costantini P Druillennec S Hoebeke J Briand JP The HIV-1 viral protein R induces apoptosis via a direct effect on the mitochondrial permeability transition pore J Exp Med 2000 191 33 46 10.1084/jem.191.1.33 10620603 PMC2195797

[B14] Stewart SA Poon B Jowett JB Chen IS Human immunodeficiency virus type 1 Vpr induces apoptosis following cell cycle arrest J Virol 1997 71 5579 5592 9188632 10.1128/jvi.71.7.5579-5592.1997 PMC191800

[B15] Di Marzio P Choe S Ebright M Knoblauch R Landau NR Mutational analysis of cell cycle arrest, nuclear localization and virion packaging of human immunodeficiency virus type 1 Vpr J Virol 1995 69 7909 7916 7494303 10.1128/jvi.69.12.7909-7916.1995 PMC189735

[B16] Jacquot G Le Rouzic E David A Mazzolini J Bouchet J Bouaziz S Niedergang F Pancino G Benichou S Localization of HIV-1 Vpr to the nuclear envelope: impact on Vpr functions and virus replication in macrophages Retrovirology 2007 4 84 10.1186/1742-4690-4-84 18039376 PMC2211753

[B17] Jenkins Y McEntee M Weis K Greene WC Characterization of HIV-1 vpr nuclear import: analysis of signals and pathways J Cell Biol 1998 143 875 885 10.1083/jcb.143.4.875 9817747 PMC2132945

[B18] Kamata M Aida Y Two putative alpha-helical domains of human immunodeficiency virus type 1 Vpr mediate nuclear localization by at least two mechanisms J Virol 2000 74 7179 7186 10.1128/JVI.74.15.7179-7186.2000 10888660 PMC112238

[B19] Nakazawa J Watanabe N Imoto M Osada H Mutational analysis of growth arrest and cellular localization of human immunodeficiency virus type 1 Vpr in the budding yeast, Saccharomyces cerevisiae J Gen Appl Microbiol 2005 51 245 256 10.2323/jgam.51.245 16205032

[B20] Sherman MP de Noronha CM Heusch MI Greene S Greene WC Nucleocytoplasmic shuttling by human immunodeficiency virus type 1 Vpr J Virol 2001 75 1522 1532 10.1128/JVI.75.3.1522-1532.2001 11152524 PMC114057

[B21] Vodicka MA Koepp DM Silver PA Emerman M HIV-1 Vpr interacts with the nuclear transport pathway to promote macrophage infection Genes Dev 1998 12 175 185 10.1101/gad.12.2.175 9436978 PMC316441

[B22] Mahalingam S Ayyavoo V Patel M Kieber-Emmons T Weiner DB Nuclear import, virion incorporation, and cell cycle arrest/differentiation are mediated by distinct functional domains of human immunodeficiency virus type 1 Vpr J Virol 1997 71 6339 6347 9261351 10.1128/jvi.71.9.6339-6347.1997 PMC191907

[B23] Morellet N Roques BP Bouaziz S Structure-function relationship of Vpr: biological implications Curr HIV Res 2009 7 184 210 10.2174/157016209787581490 19275588

[B24] Pandey RC Datta D Mukerjee R Srinivasan A Mahalingam S Sawaya BE HIV-1 Vpr: a closer look at the multifunctional protein from the structural perspective Curr HIV Res 2009 7 114 128 10.2174/157016209787581508 19275580

[B25] Caly L Saksena NK Piller SC Jans DA Impaired nuclear import and viral incorporation of Vpr derived from a HIV long-term non-progressor Retrovirology 2008 5 67 10.1186/1742-4690-5-67 18638397 PMC2515335

[B26] Depienne C Roques P Creminon C Fritsch L Casseron R Dormont D Dargemont C Benichou S Cellular distribution and karyophilic properties of matrix, integrase, and Vpr proteins from the human and simian immunodeficiency viruses Exp Cell Res 2000 260 387 395 10.1006/excr.2000.5016 11035935

[B27] Waldhuber MG Bateson M Tan J Greenway AL McPhee DA Studies with GFP-Vpr fusion proteins: induction of apoptosis but ablation of cell-cycle arrest despite nuclear membrane or nuclear localization Virology 2003 313 91 104 10.1016/S0042-6822(03)00258-7 12951024

[B28] Jacquot G Le Rouzic E Maidou-Peindara P Maizy M Lefrere JJ Daneluzzi V Monteiro-Filho CM Hong D Planelles V Morand-Joubert L Benichou S Characterization of the molecular determinants of primary HIV-1 Vpr proteins: impact of the Q65R and R77Q substitutions on Vpr functions PLoS One 2009 4 e7514 10.1371/journal.pone.0007514 19838296 PMC2759284

[B29] Le Rouzic E Mousnier A Rustum C Stutz F Hallberg E Dargemont C Benichou S Docking of HIV-1 Vpr to the nuclear envelope is mediated by the interaction with the nucleoporin hCG1 J Biol Chem 2002 277 45091 45098 10.1074/jbc.M207439200 12228227

[B30] Wang L Mukherjee S Narayan O Zhao LJ Characterization of a leucine-zipper-like domain in Vpr protein of human immunodeficiency virus type 1 Gene 1996 178 7 13 10.1016/0378-1119(96)00312-5 8921884

[B31] Gendelman HE Baca LM Husayni H Turpin JA Skillman D Kalter DC Orenstein JM Hoover DL Meltzer MS Macrophage-HIV interaction: viral isolation and target cell tropism AIDS 1990 4 221 228 10.1097/00002030-199003000-00007 2112397

[B32] Lyerly HK Matthews TJ Langlois AJ Bolognesi DP Weinhold KJ Human T-cell lymphotropic virus IIIB glycoprotein (gp120) bound to CD4 determinants on normal lymphocytes and expressed by infected cells serves as target for immune attack Proc Natl Acad Sci USA 1987 84 4601 4605 10.1073/pnas.84.13.4601 3037522 PMC305138

[B33] He J Chen Y Farzan M Choe H Ohagen A Gartner S Busciglio J Yang X Hofmann W Newman W CCR3 and CCR5 are co-receptors for HIV-1 infection of microglia Nature 1997 385 645 649 10.1038/385645a0 9024664

[B34] Wu DT Woodman SE Weiss JM McManus CM D'Aversa TG Hesselgesser J Major EO Nath A Berman JW Mechanisms of leukocyte trafficking into the CNS J Neurovirol 2000 6 Suppl 1 S82 85 10871769

[B35] Wheeler ED Achim CL Ayyavoo V Immunodetection of human immunodeficiency virus type 1 (HIV-1) Vpr in brain tissue of HIV-1 encephalitic patients J Neurovirol 2006 12 200 210 10.1080/13550280600827377 16877301

[B36] Churchill MJ Gorry PR Cowley D Lal L Sonza S Purcell DF Thompson KA Gabuzda D McArthur JC Pardo CA Wesselingh SL Use of laser capture microdissection to detect integrated HIV-1 DNA in macrophages and astrocytes from autopsy brain tissues J Neurovirol 2006 12 146 152 10.1080/13550280600748946 16798676

[B37] Churchill MJ Wesselingh SL Cowley D Pardo CA McArthur JC Brew BJ Gorry PR Extensive astrocyte infection is prominent in human immunodeficiency virus-associated dementia Ann Neurol 2009 66 253 258 10.1002/ana.21697 19743454

[B38] Takahashi K Wesselingh SL Griffin DE McArthur JC Johnson RT Glass JD Localization of HIV-1 in human brain using polymerase chain reaction/in situ hybridization and immunocytochemistry Ann Neurol 1996 39 705 711 10.1002/ana.410390606 8651642

[B39] Kanmogne GD Grammas P Kennedy RC Analysis of human endothelial cells and cortical neurons for susceptibility to HIV-1 infection and co-receptor expression J Neurovirol 2000 6 519 528 10.3109/13550280009091952 11175324

[B40] Meucci O Fatatis A Simen AA Bushell TJ Gray PW Miller RJ Chemokines regulate hippocampal neuronal signaling and gp120 neurotoxicity Proc Natl Acad Sci USA 1998 95 14500 14505 10.1073/pnas.95.24.14500 9826729 PMC24402

[B41] Piller SC Ewart GD Premkumar A Cox GB Gage PW Vpr protein of human immunodeficiency virus type 1 forms cation-selective channels in planar lipid bilayers Proc Natl Acad Sci USA 1996 93 111 115 10.1073/pnas.93.1.111 8552585 PMC40188

[B42] Rom I Deshmane SL Mukerjee R Khalili K Amini S Sawaya BE HIV-1 Vpr deregulates calcium secretion in neural cells Brain Res 2009 1275 81 86 19328187 10.1016/j.brainres.2009.03.024 PMC2692350

[B43] Matz MV Fradkov AF Labas YA Savitsky AP Zaraisky AG Markelov ML Lukyanov SA Fluorescent proteins from nonbioluminescent Anthozoa species Nat Biotechnol 1999 17 969 973 10.1038/13657 10504696

[B44] Snyder A Alsauskas Z Gong P Rosenstiel PE Klotman ME Klotman PE Ross MJ FAT10: a novel mediator of Vpr-induced apoptosis in human immunodeficiency virus-associated nephropathy J Virol 2009 83 11983 11988 10.1128/JVI.00034-09 19726511 PMC2772664

[B45] Nakamura T Suzuki H Okamoto T Kotani S Atsuji Y Tanaka T Ito Y Recombinant Vpr (rVpr) causes augmentation of HIV-1 p24 Ag level in U1 cells through its ability to induce the secretion of TNF Virus Res 2002 90 263 268 10.1016/S0168-1702(02)00230-7 12457980

[B46] de Noronha CM Sherman MP Lin HW Cavrois MV Moir RD Goldman RD Greene WC Dynamic disruptions in nuclear envelope architecture and integrity induced by HIV-1 Vpr Science 2001 294 1105 1108 10.1126/science.1063957 11691994

[B47] Shinya E Owaki A Shimizu M Takeuchi J Kawashima T Hidaka C Satomi M Watari E Sugita M Takahashi H Endogenously expressed HIV-1 nef down-regulates antigen-presenting molecules, not only class I MHC but also CD1a, in immature dendritic cells Virology 2004 326 79 89 10.1016/j.virol.2004.06.004 15262497

[B48] Laguette N Benichou S Basmaciogullari S Human immunodeficiency virus type 1 Nef incorporation into virions does not increase infectivity J Virol 2009 83 1093 1104 10.1128/JVI.01633-08 18987145 PMC2612363

[B49] Campbell TD Khan M Huang MB Bond VC Powell MD HIV-1 Nef protein is secreted into vesicles that can fuse with target cells and virions Ethn Dis 2008 18 S2 14-19 18646314 PMC3418053

[B50] Balasubramanyam A Mersmann H Jahoor F Phillips TM Sekhar RV Schubert U Brar B Iyer D Smith EO Takahashi H Effects of transgenic expression of HIV-1 Vpr on lipid and energy metabolism in mice Am J Physiol Endocrinol Metab 2007 292 E40 48 16882932 10.1152/ajpendo.00163.2006

[B51] Gonzalez-Scarano F Martin-Garcia J The neuropathogenesis of AIDS Nat Rev Immunol 2005 5 69 81 10.1038/nri1527 15630430

[B52] Kaul M Garden GA Lipton SA Pathways to neuronal injury and apoptosis in HIV-associated dementia Nature 2001 410 988 994 10.1038/35073667 11309629

[B53] Maingat F Halloran B Acharjee S van Marle G Church D Gill MJ Uwiera RR Cohen EA Meddings J Madsen K Power C Inflammation and epithelial cell injury in AIDS enteropathy: involvement of endoplasmic reticulum stress FASEB J 2011 25 7 2211 20 10.1096/fj.10-175992 21427211 PMC3114526

[B54] Shrivastav S Kino T Cunningham T Ichijo T Schubert U Heinklein P Chrousos GP Kopp JB Human immunodeficiency virus (HIV)-1 viral protein R suppresses transcriptional activity of peroxisome proliferator-activated receptor {gamma} and inhibits adipocyte differentiation: implications for HIV-associated lipodystrophy Mol Endocrinol 2008 22 234 247 17932108 10.1210/me.2007-0124 PMC2234580

[B55] He J Choe S Walker R Di Marzio P Morgan DO Landau NR Human immunodeficiency virus type 1 viral protein R (Vpr) arrests cells in the G2 phase of the cell cycle by inhibiting p34cdc2 activity J Virol 1995 69 6705 6711 7474080 10.1128/jvi.69.11.6705-6711.1995 PMC189580

[B56] Zhao RY Li G Bukrinsky MI Vpr-Host Interactions During HIV-1 Viral Life Cycle J Neuroimmune Pharmacol 2011 6 2 216 29 10.1007/s11481-011-9261-z 21318276 PMC5482210

[B57] Nonaka M Hashimoto Y Takeshima SN Aida Y The human immunodeficiency virus type 1 Vpr protein and its carboxy-terminally truncated form induce apoptosis in tumor cells Cancer Cell Int 2009 9 20 10.1186/1475-2867-9-20 19674438 PMC2735735

[B58] Fritz JV Didier P Clamme JP Schaub E Muriaux D Cabanne C Morellet N Bouaziz S Darlix JL Mely Y de Rocquigny H Direct Vpr-Vpr interaction in cells monitored by two photon fluorescence correlation spectroscopy and fluorescence lifetime imaging Retrovirology 2008 5 87 10.1186/1742-4690-5-87 18808682 PMC2562391

[B59] Thotala D Schafer EA Tungaturthi PK Majumder B Janket ML Wagner M Srinivasan A Watkins S Ayyavoo V Structure-functional analysis of human immunodeficiency virus type 1 (HIV-1) Vpr: role of leucine residues on Vpr-mediated transactivation and virus replication Virology 2004 328 89 100 10.1016/j.virol.2004.07.013 15380361

[B60] Zhou Y Lu Y Ratner L Arginine residues in the C-terminus of HIV-1 Vpr are important for nuclear localization and cell cycle arrest Virology 1998 242 414 424 10.1006/viro.1998.9028 9514978

[B61] Bolton DL Lenardo MJ Vpr cytopathicity independent of G2/M cell cycle arrest in human immunodeficiency virus type 1-infected CD4+ T cells J Virol 2007 81 8878 8890 10.1128/JVI.00122-07 17553871 PMC1951439

[B62] Muthumani K Hwang DS Dayes NS Kim JJ Weiner DB The HIV-1 accessory gene vpr can inhibit antigen-specific immune function DNA Cell Biol 2002 21 689 695 10.1089/104454902760330237 12396612

[B63] Henklein P Bruns K Sherman MP Tessmer U Licha K Kopp J de Noronha CM Greene WC Wray V Schubert U Functional and structural characterization of synthetic HIV-1 Vpr that transduces cells, localizes to the nucleus, and induces G2 cell cycle arrest J Biol Chem 2000 275 32016 32026 10903315 10.1074/jbc.M004044200

[B64] Lahti AL Manninen A Saksela K Regulation of T cell activation by HIV-1 accessory proteins: Vpr acts via distinct mechanisms to cooperate with Nef in NFAT-directed gene expression and to promote transactivation by CREB Virology 2003 310 190 196 10.1016/S0042-6822(03)00164-8 12788643

[B65] Xiao Y Chen G Richard J Rougeau N Li H Seidah NG Cohen EA Cell-surface processing of extracellular human immunodeficiency virus type 1 Vpr by proprotein convertases Virology 2008 372 384 397 10.1016/j.virol.2007.10.036 18061232 PMC3955186

[B66] Hoshino S Sun B Konishi M Shimura M Segawa T Hagiwara Y Koyanagi Y Iwamoto A Mimaya J Terunuma H Vpr in plasma of HIV type 1-positive patients is correlated with the HIV type 1 RNA titers AIDS Res Hum Retroviruses 2007 23 391 397 10.1089/aid.2006.0124 17411372

[B67] Levy DN Refaeli Y MacGregor RR Weiner DB Serum Vpr regulates productive infection and latency of human immunodeficiency virus type 1 Proc Natl Acad Sci USA 1994 91 10873 10877 10.1073/pnas.91.23.10873 7971975 PMC45128

[B68] Bouhamdan M Benichou S Rey F Navarro JM Agostini I Spire B Camonis J Slupphaug G Vigne R Benarous R Sire J Human immunodeficiency virus type 1 Vpr protein binds to the uracil DNA glycosylase DNA repair enzyme J Virol 1996 70 697 704 8551605 10.1128/jvi.70.2.697-704.1996 PMC189869

[B69] Nilsen H Otterlei M Haug T Solum K Nagelhus TA Skorpen F Krokan HE Nuclear and mitochondrial uracil-DNA glycosylases are generated by alternative splicing and transcription from different positions in the UNG gene Nucleic Acids Res 1997 25 750 755 10.1093/nar/25.4.750 9016624 PMC146498

[B70] Zhao RY Li G Bukrinsky MI Vpr-Host Interactions During HIV-1 Viral Life Cycle J Neuroimmune Pharmacol 2011 6 216 229 10.1007/s11481-011-9261-z 21318276 PMC5482210

[B71] Jones GJ Barsby NL Cohen EA Holden J Harris K Dickie P Jhamandas J Power C HIV-1 Vpr causes neuronal apoptosis and in vivo neurodegeneration J Neurosci 2007 27 3703 3711 10.1523/JNEUROSCI.5522-06.2007 17409234 PMC6672409

[B72] Kozak M At least six nucleotides preceding the AUG initiator codon enhance translation in mammalian cells J Mol Biol 1987 196 947 950 10.1016/0022-2836(87)90418-9 3681984

[B73] Jenkins Y Sanchez PV Meyer BE Malim MH Nuclear export of human immunodeficiency virus type 1 Vpr is not required for virion packaging J Virol 2001 75 8348 8352 10.1128/JVI.75.17.8348-8352.2001 11483780 PMC115079

[B74] Sherman MP de Noronha CM Pearce D Greene WC Human immunodeficiency virus type 1 Vpr contains two leucine-rich helices that mediate glucocorticoid receptor coactivation independently of its effects on G(2) cell cycle arrest J Virol 2000 74 8159 8165 10.1128/JVI.74.17.8159-8165.2000 10933727 PMC112350

[B75] Lai M Chen J The role of Vpr in HIV-1 disease progression is independent of its G2 arrest induction function Cell Cycle 2006 5 2275 2280 10.4161/cc.5.19.3317 16969134

[B76] Zhao LJ Wang L Mukherjee S Narayan O Biochemical mechanism of HIV-1 Vpr function. Oligomerization mediated by the N-terminal domain J Biol Chem 1994 269 32131 32137 7798208

[B77] Krichevsky A Graessmann A Nissim A Piller SC Zakai N Loyter A Antibody fragments selected by phage display against the nuclear localization signal of the HIV-1 Vpr protein inhibit nuclear import in permeabilized and intact cultured cells Virology 2003 305 77 92 10.1006/viro.2002.1765 12504543

[B78] Zhu Y Gelbard HA Roshal M Pursell S Jamieson BD Planelles V Comparison of cell cycle arrest, transactivation, and apoptosis induced by the simian immunodeficiency virus SIVagm and human immunodeficiency virus type 1 vpr genes J Virol 2001 75 3791 3801 10.1128/JVI.75.8.3791-3801.2001 11264368 PMC114870

[B79] Andersen JL Zimmerman ES DeHart JL Murala S Ardon O Blackett J Chen J Planelles V ATR and GADD45alpha mediate HIV-1 Vpr-induced apoptosis Cell Death Differ 2005 12 326 334 10.1038/sj.cdd.4401565 15650754

[B80] Yedavalli VS Shih HM Chiang YP Lu CY Chang LY Chen MY Chuang CY Dayton AI Jeang KT Huang LM Human immunodeficiency virus type 1 Vpr interacts with antiapoptotic mitochondrial protein HAX-1 J Virol 2005 79 13735 13746 10.1128/JVI.79.21.13735-13746.2005 16227293 PMC1262574

[B81] Yao XJ Subbramanian RA Rougeau N Boisvert F Bergeron D Cohen EA Mutagenic analysis of human immunodeficiency virus type 1 Vpr: role of a predicted N-terminal alpha-helical structure in Vpr nuclear localization and virion incorporation J Virol 1995 69 7032 7044 7474123 10.1128/jvi.69.11.7032-7044.1995 PMC189623

